# Biliary peritonitis caused by a leaking T-tube fistula disconnected at the point of contact with the anterior abdominal wall: a case report

**DOI:** 10.1186/1752-1947-2-302

**Published:** 2008-09-16

**Authors:** Marko Nikolić, Alan Karthikesalingam, Senthil Nachimuthu, Tjun Y Tang, Adrian M Harris

**Affiliations:** 1 Cambridge University Hospitals NHS Foundation Trust, Hills Road, Cambridge CB2 2QQ, UK; 2 Department of General Surgery, Hinchingbrooke Hospital NHS Trust, Hinchingbrooke Park, Huntingdon PE29 6NT, UK

## Abstract

**Introduction:**

Operations on the common bile duct may lead to potentially serious complications such as biliary peritonitis. T-tube insertion is performed to reduce the risk of this occurring postoperatively. Biliary leakage at the point of insertion into the common bile duct, or along the fistula, can sometimes occur after T-tube removal and this has been reported extensively in the literature. We report a case where the site at which the T-tube fistula leaked proved to be the point of contact between the fistula and the anterior abdominal wall, a previously unreported complication.

**Case presentation:**

A 36-year-old sub-Saharan African woman presented with gallstone-induced pancreatitis and, once her symptoms settled, laparoscopic cholecystectomy was performed, common bile duct stones were removed and a T-tube was inserted. Three weeks later, T-tube removal led to biliary peritonitis due to the disconnection of the T-tube fistula which was recannulated laparoscopically using a Latex drain.

**Conclusion:**

This case highlights a previously unreported mechanism for bile leak following T-tube removal caused by detachment of a fistula tract at its contact point with the anterior abdominal wall. Hepatobiliary surgeons should be aware of this mechanism of biliary leakage and the use of laparoscopy to recannulate the fistula.

## Introduction

The placement of a T-tube to drain the biliary system is a widely used alternative to primary closure of choledochotomy following Common Bile Duct (CBD) exploration, especially in a non-dilated system. T-tubes are used to ensure decompression of the biliary tree by creating a fibrous fistula to the anterior abdominal wall. This permits healing of the choledochotomy incision and reduces the risk of bile leak and stricture formation [1,2]. A small bile discharge from the dermal ostium of the fistula may still be observed but usually stops within 24 hours after removal of the tube without causing biliary peritonitis [3]. As long as there is no distal CBD obstruction, normal intra-abdominal pressure will cause compression and obliteration of the fistula lumen. We describe a case where the fistula tract failed to adhere to the anterior abdominal wall, causing a leak after removal of the T-tube.

## Case presentation

A 36-year-old sub-Saharan African woman presented to the Accident and Emergency department with a 7-hour history of vomiting and central abdominal pain radiating to the back. There were no respiratory, cardiovascular or urinary symptoms, and past medical history was unremarkable. The blood results included an amylase of 3070 U/litre and an abdominal ultrasound showed multiple tiny gallstones confined to a thin-walled gallbladder with normal pancreas, liver, kidneys and spleen. A diagnosis of gallstone-induced pancreatitis was made and laparoscopic cholecystectomy was performed 5 days later, once her symptoms had settled. An on-table cholangiogram demonstrated a filling defect at the distal end of the CBD with no duodenal filling. Laparoscopic CBD exploration was undertaken and two stones were removed from the distal CBD using a Dormia basket through the choledochoscope. A Latex 12-Fr T-tube was inserted into the CBD at the end of the procedure. The patient made an uneventful recovery postoperatively and was discharged with the T-tube spigotted and left *in situ*.

**Figure 1:**
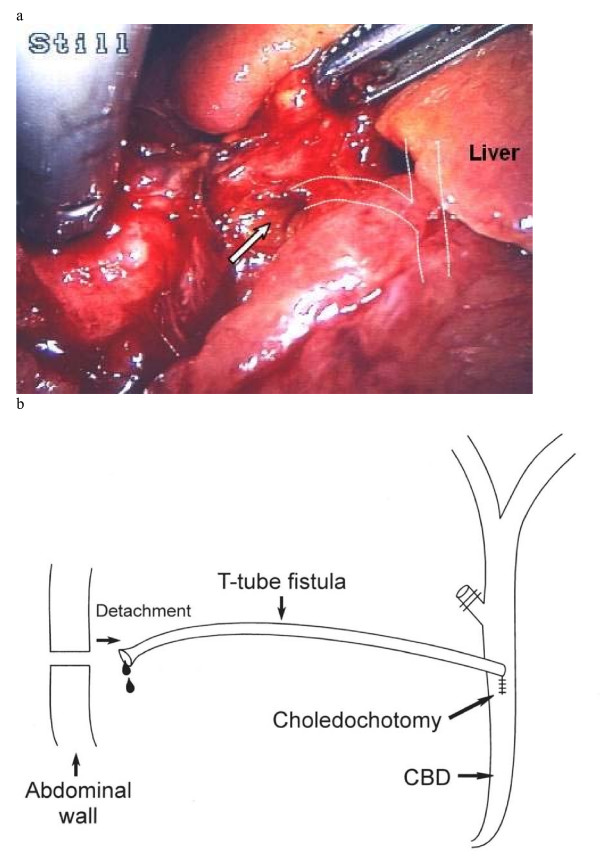
**(a) T-tube fistula tract opening.** Intraoperative laparoscopic photograph illustrating opening to T-tube fistula tract (arrow) with diagrammatic representation of relation to biliary anatomy. (b) Diagram of fistula pathway and leak mechanism.

**Figure 2:**
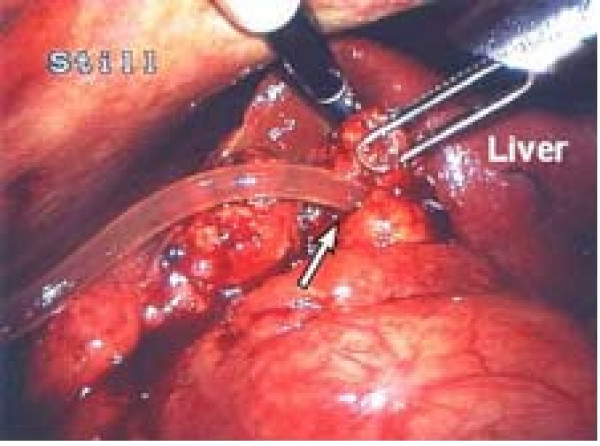
**Cannulation of T-tube fistula.** Intraoperative laparoscopic photograph illustrating cannulation of T-tube fistula tract with 10-Fr Latex drain.

A T-tube cholangiogram 3 weeks later excluded any bile duct obstruction or leakage and the T-tube was therefore removed without difficulty. However, the patient soon started vomiting and complained of increasingly severe right upper quadrant abdominal pain. Following overnight observation, ultrasonography for suspected bile leak was inconclusive. Biliary peritonitis was clinically suspected and an emergency diagnostic laparoscopy was performed. This revealed that the fistula had become disconnected at the point of contact with the anterior abdominal wall (Fig. 1a,b). Bile was clearly visible draining from the fistula opening (arrowed). The whole length of the fistula was inspected and no other leak was found; the proximal junction with the CBD was intact. The distal fistula was recannulated with a 10 Fr Latex drain (Fig. 2) and bile was observed to be draining freely from it. Following an uneventful recovery, cholangiography of the cannulated tract with the Latex drain *in-situ *was repeated after 5 weeks and no dye was able to pass down it. The tract was therefore presumed to have closed. The Latex drain was removed 24 hours later with good recovery to date and no further complications.

## Discussion

Biliary peritonitis is regarded as a rare but serious complication of elective T-tube removal after CBD exploration. Incidence reported in the literature varies from 0.8 to 5% in elective removal of T-tubes, rising to 24% in cases of liver transplants [3].

Historically, a latex T-tube has always been used during open exploration, specifically to encourage a vigorous inflammatory reaction around it causing formation of a biliary fistula. This makes T-tube removal much safer by reducing the potential for intraperitoneal bile leak. The fistula closes rapidly after removal of the T-tube as long as there is no distal CBD obstruction. More recently, silicone-coated or polyethylene T-tubes have become available, but these are less irritant and the resulting fistula tends to be less mature, increasing the risk of a leak after T-tube removal. We do not recommend use of these newer T-tubes after CBD exploration unless the patient has a latex allergy.

This case is novel since the site of the bile leak was distal, at the point of contact between fistula and anterior abdominal wall. Usually biliary leakage occurs through lack of complete fibrous T-tube fistula formation or through proximal fistula disruption during the removal process [4]. Identification of the location of the leak as described was important for three reasons: first, it provided an accurate diagnosis; second, it confirmed that the usual leak point (i.e. the junction between fistula and CBD) was intact and did not therefore require a further T-tube placement, and third, it allowed a simple therapeutic manoeuvre by re-intubating the fistula opening.

It may be suggested that the fistula was disrupted from the abdominal wall during insufflation at the time of laparoscopy. However, this does not explain the clinical presentation *before *laparoscopy. We knew there was no leak before T-tube removal because of a normal T-tube cholangiogram and lack of abdominal symptoms. The patient suffered upper abdominal pain soon after removal of the T-tube, developing biliary peritonitis along with raised inflammatory markers (white cell count and C-reactive protein). This indicates a leak at the time of T-tube removal which was subsequently confirmed at laparoscopy with a pool of bile on the surface of the abdominal viscera. Bile was clearly observed emanating directly from the distal fistula opening.

The literature has been reviewed in view of factors which affect the risk of biliary leakage.

### T-tubes versus choledochorrhaphy

The first option is to avoid T-tube insertion altogether and perform a choledochorrhaphy (primary closure of the choledochotomy). In the past, this was rarely advised as it was thought to increase the risk of stricture formation and prevent postoperative CBD decompression. However, research has recently suggested that primary closure may be as safe as T-tube usage [5], although it should be avoided if the CBD is not significantly dilated. The other benefit of T-tubes is the ease of postoperative visualisation of retained CBD stones (T-tube cholangiogram).

### Duration of T-tube insertion

Many factors may affect the risk of symptomatic bile leakage following T-tube removal. Ellis [2] originally suggested that T-tubes should be removed 10 days after operation. It has been suggested that leaving T-tubes *in situ *for longer periods allows maturation of the temporary biliary cutaneous fistula, thus potentially reducing the risk of leakage [4]. However, there is no experimental evidence to prove this hypothesis. Indeed, one study has shown that leaving T-tubes *in situ *for longer periods, such as 1 month postoperatively does not provide protection against increased rates of bile leakage [1]. In this case, the T-tube was removed after 3 weeks, in line with common practice in the UK.

### T-tube material

Experimental evidence demonstrates that the material used for manufacturing T-tubes affects the quality of fibrous fistula formed [6,7]. This finding is supported by clinical evidence that polyvinyl chloride (PVC) or hypoallergenic latex T-tubes (such as those coated with silicon) increase rates of biliary peritonitis compared to red rubber or normal latex T-tubes, as the former take longer to form a mature tract [8]. In our case, therefore, the standard latex T-tube used is unlikely to be of aetiological significance.

### Immune system

The hypothesis that an increased inflammatory response leads to the formation of a stronger fistulous tract may explain the increased rates of symptomatic bile leaks in immunocompromised patients, such as those undergoing liver transplantation [3]. In our case, there was no past medical history of diabetes or steroid use and no medical evidence of occult immunosuppressive pathologies, although an HIV test was not performed.

### T-tube morphology

**Figure 3:**
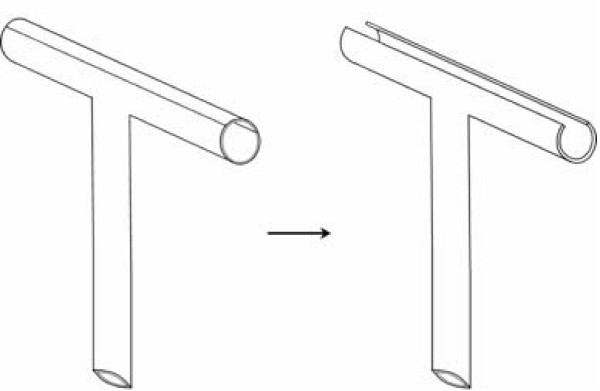
**T-tube morphology.** A gutter is cut out of the cross arm to lower resistance during T-tube removal and thus reduce the risk of traumatic fistula disruption.

It has been suggested that the morphology of the T-tube or its placement could reduce leakage [2], although other authors have pointed out that there is no experimental evidence for this theory. Figure 3 illustrates the morphology of the T-tube used in this patient, designed to minimise trauma during T-tube removal and thus potentially the risk of biliary leakage [9]. Some authorities recommend cutting a notch in the short 'crossbar', opposite the drainage tube, to further facilitate removal by allowing the two 'wings' to fold more easily. If this is done, care must be taken not to make the resulting bridge of material too thin as the wings may then detach during the removal process.

### T-tube removal technique

Goodwin *et al*. [10] reported a significant reduction in bile leakage and subsequent biliary peritonitis after T-tube removal in liver transplant patients when the tube was removed along a wire (Seldinger method). This technique is generally only recommended in high-risk patients in whom bile leakage is anticipated following T-tube removal, especially in immunocompromised patients following liver transplantation.

## Conclusion

This case and our review of the literature highlight a previously unreported mechanism for bile leak following T-tube removal caused by dehiscence of a fistula tract at its contact point with the anterior abdominal wall. Hepatobiliary surgeons should be aware of this mechanism of biliary leakage and the use of laparoscopy to recannulate the fistula with a satisfactory outcome.

## Consent

Written informed consent was obtained from the patient for publication of this case report and any accompanying images. A copy of the written consent is available for review by the Editor-in-Chief of this journal.

## Competing interests

The authors declare that they have no competing interests.

## Authors' contributions

MN and AK were involved in design of the case report, drafted the manuscript and performed critical literature review. AH, SN and TT conceived the original idea of the case report, conducted the operations detailed and have been involved in critically revising the manuscript.
